# Occupational Exposure to Metal-Based Nanomaterials: A Possible Relationship between Chemical Composition and Oxidative Stress Biomarkers

**DOI:** 10.3390/antiox13060676

**Published:** 2024-05-31

**Authors:** Valeria Bellisario, Giacomo Garzaro, Giulia Squillacioti, Marco Panizzolo, Federica Ghelli, Giuseppe Mariella, Roberto Bono, Irina Guseva Canu, Enrico Bergamaschi

**Affiliations:** 1 Department of Public Health and Pediatrics, University of Turin, 10126 Turin, Italy; valeria.bellisario@unito.it (V.B.); giacomo.garzaro@unito.it (G.G.); giulia.squillacioti@unito.it (G.S.); marco.panizzolo@unito.it (M.P.); federica.ghelli@unito.it (F.G.); giuseppe.mariella@unito.it (G.M.); enrico.bergamaschi@unito.it (E.B.); 2 Department of Occupational and Environmental Health, Center for Primary Care and Public Health (Unisanté), University of Lausanne, 1010 Lausanne, Switzerland; irina.guseva-canu@unisante.ch

**Keywords:** occupational air pollution exposure, NM biomonitoring, NM occupational exposure, nanosized metal, body burden, oxidative imbalance, antioxidant defenses

## Abstract

Nanomaterials (NMs) are in high demand for a wide range of practical applications; however, comprehensively understanding the toxicity of these materials is a complex challenge, due to the limited availability of epidemiological evidence on the human health effects arising from workplace exposures. The aim of this work is to assess whether and how urinary metal concentrations could be reliable and useful in NM biomonitoring. In the framework of “NanoExplore Project” [EU LIFE17 Grant ENV/GR/000285], 43 not-exposed subjects and 40 exposed workers were recruited to measure exposure to NMs (PCN and LDSA) in the proximity of the workstations and biological biomarkers (urinary metal concentrations—Aluminum (Al), Silica (Si), Titanium (Ti), and Chromium (Cr); urinary OS biomarkers—TAP, Isop, and MDA). The results showed that Si and Ti were directly associated with NM exposure (both PCN and LDSA), as well as with OS biomarkers, especially in exposed workers. Moreover, the mediation analyses showed that Si could account for about 2.8% in the relationship between LDSA and OS biomarkers, possibly by decreasing OS antioxidant defenses in exposed people. In conclusion, our study provides evidence that occupational exposure to mixtures containing NMs can represent an underestimated hazard for exposed people, increasing the body burden and the oxidative balance.

## 1. Introduction

Nanomaterials (NMs) have emerged as an exciting class of materials that are in high demand for a wide range of practical applications, with an approximate annual production volume of NMs reaching 60,000 tons [1,2,3]. As an excellent example of emerging nanotechnology, NMs currently show great potential and improved performances [4] in scratch-free paints, surface coatings, electronics, cosmetics, environmental remediation, sports equipment, sensors, and energy storage devices [5]. According to the European Commission, a nanomaterial is a natural, incidental, or manufactured material consisting of solid particles characterized by at least (a) one or more external dimensions in the size range of 1 nm–100 nm; (b) an elongated shape, such as a rod, fiber, or tube, where two external dimensions are smaller than 1 nm and the other dimension is larger than 100 nm; and (c) a plate-like shape, where one external dimension is smaller than 1 nm and the other dimensions are larger than 100 nm [6]. Carbon, metal, metal oxides, or organic substances are special classes of NMs, and the release of nanoparticles originating from the handling of micron-sized materials may be substantial in some occupational scenarios [7,8].

While the number of NM types and applications continues to increase, the knowledge on the health effects of nanoparticles exposure is still limited, even if the number of efforts aimed at determining the health risks associated with NM exposure continues to grow. As these nanoparticles are intentionally engineered to interact with cells, it is fundamental to ensure that these enhancements are not causing any adverse effects, undergoing biodegradation, or causing damage in the cellular environment [9]. For example, biodegraded nanoparticles may accumulate within cells and lead to intracellular changes, such as disruption of organelle integrity or gene alterations [10]. In vitro studies, using different cell lines and protocols, of chemically different nanoparticles have revealed a potential hazard; however, these surrogate models could only estimate what could occur after the interactions with biological systems. Moreover, these studies include a wide range of nanoparticle concentrations, making it difficult to determine whether the toxicity endpoints are relevant for human beings [11]. While in vitro nanoparticle applications allow less stringent toxicological characterization, in vivo studies of NMs require thorough understanding of the kinetics and toxicology of the particles. Although the toxicology of metals is a mature science, many interactions of metal nanoparticles with biological systems are still controversial.

Metal NMs interact with complex biological systems, such as the human body, and many epidemiological studies [8,12,13,14,15] have addressed the general role of these nanoparticles in neurodegeneration, neurotoxicity, and oxidative imbalance, as well as lung injury [16,17]. In particular, some works [17,18,19] suggest that Al could have some redox capacity and could be linked to oxidative damage, reacting with H_2_O_2_, producing Al superoxide radicals (AlO_2_•^−^), and promoting the generation of ROS. Owing to its high reactivity, Al is primarily found forming insoluble oxides, whose toxicity seems to be related to the displacement of other biological cations (Ca II, Fe II, or Mg Ife). Silica and Titanium may impair antioxidant/oxidant status and activate the immune system, which is indicative of inflammatory responses, but the mechanisms are not clearly understood [20,21,22]. Chromium (Cr III and IV) [23] could induce DNA damage, as MN or aberrations, instead of oxidative stress imbalance and inflammation.

Therefore, fully understanding the toxicity of these materials is a complex task, especially because there has been limited occupational epidemiologic evidence of human health effects from workplace exposures to NMs [24]. Possible adverse health effects can result from additional sources of metal NMs, thus increasing their body burden. This can occur especially in occupational settings, where the mass of airborne particles, instead of more appropriate metrics, such as the particle number concentration, is still considered [7,25].

In this context, we aimed to assess whether and how urinary metal concentrations, particularly Aluminum (Al), Titanium (Ti), Chromium (Cr), and Silica (Si), a metalloid, could be reliable biomarkers of exposure for NM biomonitoring. Moreover, we aimed to assess whether urinary metals are associated with oxidative stress biomarkers with a mediation and trigger role. Finally, this work assessed whether NM exposure can affect oxidative stress imbalance, in the perspectives of improving surveillance protocols and preventive strategies correlated.

## 2. Materials and Methods

### 2.1. Study Sample and Exposure to ENMs

This work is based on sampling and data gathered in the framework of the multicenter prospective cohort study “NanoExplore Project” [EU LIFE17 Grant ENV/GR/000285] [25], which aimed to investigate the association between occupational exposure to NMs and different biomarkers in workers within the European Union and Switzerland. In this work, within this main and general aim, we assessed the association between nanoparticle exposure and some urinary metals and a metalloid excreted with urine, namely Aluminum (Al), Titanium (Ti), and Chromium (Cr) and Silica (Si).

The companies were enrolled in each country based on confirmed prior knowledge of their activities related to the handling of NMs. A preliminary visit was conducted in each of the enrolled companies by the company’s environmental health and safety manager and an occupational hygienist associated with the NanoExplore study. Workers who had been previously identified as potentially exposed were invited to participate in the study. We analyzed 40 workers recruited in 4 industrial plants handling mixtures of materials containing a fraction of sub-micrometric and nanosized metal oxides (e.g., Alumina—Al_2_O_3_, Titanium dioxide—TiO_2_, Chromium ores, Siliceous sands—SiO_2_) for the production of paints, coatings, or construction chemicals. Furthermore, 43 people not exposed to chemicals and dusts were enrolled as a control group. All the subjects provided written informed consent to participate. The harmonized protocol, the cohort characteristics, the recruitment procedure, the study design, and the preliminary results have been reported elsewhere [25,26,27].

Exposure to airborne nanoparticles was measured by portable nanoparticle counters “DiSCminiTM” (Testo, Mönchaltorf, Switzerland) placed in the proximity of the workstations for two up to four consecutive working days, as already described [25,26]. The DiSCmini allowed measurements to be obtained based on two different metrics: (1) the particle number concentration (PCN, expressed as number of particles/cm^3^) and (2) the lung-deposited surface area (LDSA, expressed as µm_2_/cm^3^). Exposure was defined a priori according to the working tasks, and data provided by the DiSCmini measurements were used as independent variables.

### 2.2. Biological Sampling and Biomarkers

Biological samples were collected twice, the first on the first day of the campaign week, before the shift, and the second on the fourth day, after the shift [25]. Spot urine samples were collected in the morning before the beginning of shift and were immediately aliquoted and frozen at −20 °C until analysis. Two types of biomarkers were measured in the urine samples: (1) urinary metal concentrations (Aluminum (Al), Titanium (Ti), and Chromium (Cr)), and a metalloid (Silica (Si)) and (2) urinary OS biomarkers (Isoprostane (Isop), Malondialdehyde (MDA), and Total Antioxidant Power (TAP)). The technical information on the analytical methods adopted are reported in the Supplementary Materials (Table S1). The urinary metal concentrations were determined by inductively coupled plasma mass spectrometry (ICP-MS). In accordance with the manufacturer’s instructions, the OS biomarkers were analyzed as follows: IsoP concentrations using a competitive enzyme-linked immunoassay (EA85, Oxford, MI, USA); MDA concentrations (Thiobarbituric Acid Reactive Substances assay FR40 Oxford, MI, USA) and TAP concentrations using a colorimetric assay kit (Cupric ion reducing antioxidant capacity assay TA02, Oxford, MI, USA), respectively. In addition, the urinary creatinine concentration was measured, using the kinetic Jaffé technique, to normalize the concentration of urinary biomarkers according to urinary volume (µg/mg of creatinine). Only TAP was chosen for the analysis here, mainly because this biomarker has previously resulted in being the most sensitive urinary biomarker of OS [25].

### 2.3. Statistical Analysis

**Figure 1:**
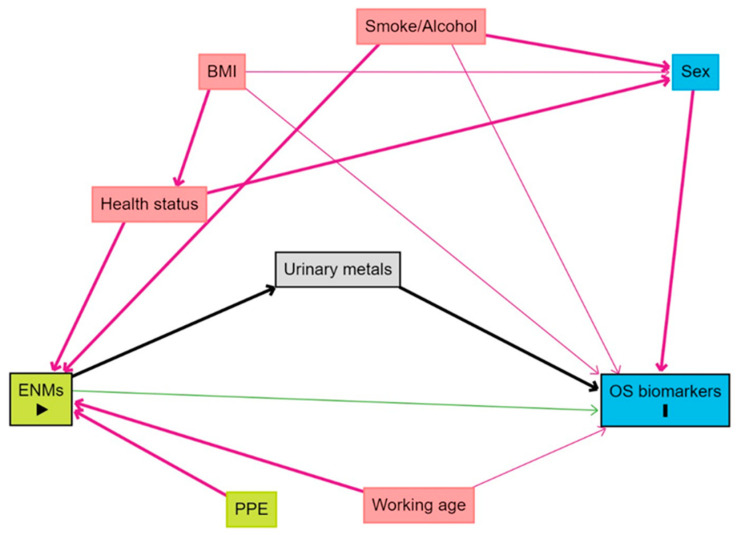
Directed acyclic graph (DAG) showing the assumed causal relationship between nanomaterial exposure and OS biomarkers. Abbreviations: exposure to nanomaterials (ENMs), personal protective equipment (PPE), body mass index (BMI).

The statistical approach has been deeply described in a previous work [25]. In the above analysis, each urinary metal biomarker was considered as independent of the other biomarkers. A directed acyclic graph (DAG) was again performed using Dagitty (^TM^), to better identify the potential confounders in the association between NM exposure, urinary metal concentrations, and OS biomarkers and, also, to identify the set of variables that should be included in the advanced models. As shown in Figure 1, these variables were working age, sex, smoking and alcohol habits, and the body mass index (BMI).

For this specific work, we decided to maintain the original exposure classification of the workers, thus analyzing only the high exposures vs. the control group, excluding the low-exposure subjects from the analysis. The descriptive general analyses were reported elsewhere [25], but for the foreplay and descriptive analyses referred to in this work, the distributions of quantitative variables were summarized with means  ±  SD, medians, and minimum and maximum values, while categorical variables were presented as number and percentage. As the statistical distribution of some quantitative parameters was found to be non-Gaussian (Kolmogorov–Smirnov test), non-parametric tests were used to assess between-group differences (Mann–Whitney U test). A two-sided *p*-value < 0.05 was considered to indicate statistical significance. Finally, associations among the exposure variables were evaluated using Pearson’s r coefficients.

The direct association between the urinary elements (Al, Si, Ti, and Cr), as the dependent variables, and the NM exposure metrics (PCN and LDSA), as the independent variables, was analyzed using a multilevel mixed-effects model, using log_10_-transformed variables. All the models considered urinary parameters as a single entity and were controlled for different potential confounders. The results were reported as relative risks (RR) with 95% confidence intervals (CIs). All the analyses were carried out using the STATA 16.1 software (StataCorp LLC: College Station, TX, USA).

Finally, the role of urinary metals as effect modifiers when assessing the association between urinary metals and OS biomarkers, represented by TAP concentrations, was explored using the medmod package (for Jamovi 2.3.28 and Rstudio 3.6 softwares), which showed the mediation estimates, as a percentage, and the individual path estimates with 95% confidence intervals (CIs).

## 3. Results

In this work, the main outcome was fixed by the analysis of the possible associations between NM exposure, represented by PCN and LDSA measurements, and some urinary biomarkers of exposure, i.e., urinary metal concentrations, and early biological effects, such as TAP concentrations. Table 1 summarizes the main characteristics of the sample extracted from the cohort described previously [25].

Non-parametric correlation analyses were performed between the exposure data, urinary concentrations of nanosized metals, and OS biomarkers. TAP was associated with Silica (*p* = 0.03), Titanium (*p* < 0.001), Chromium (*p* = 0.004), PCN (*p* < 0.001), and LDSA (*p* < 0.001). Conversely, no associations were found with the other OS biomarkers (Isop and MDA).

All the models’ results were presented (1) first in the whole sample and then (2) as stratified by exposure pattern (exposed vs. not-exposed subjects). Since the correlations did not show significant associations between the metals, the metals were analyzed one by one. We adopted mixed multilevel regression models with ID and center as random variables, and each model was adjusted according to the subjects’ personal characteristics (BMI, smoking, health score, alcohol consumption, gender), working characteristics (exposure YES/NO, employment duration, use of PPE) and the quantitative variables measured (PCN, LDSA, urinary OS biomarkers). The results were summarized according to the type of exposure metrics (PCN and LDSA), and the significant relative risk with 95% confidence intervals (CIs) was reported.

### 3.1. Exposure

**Figure 2:**
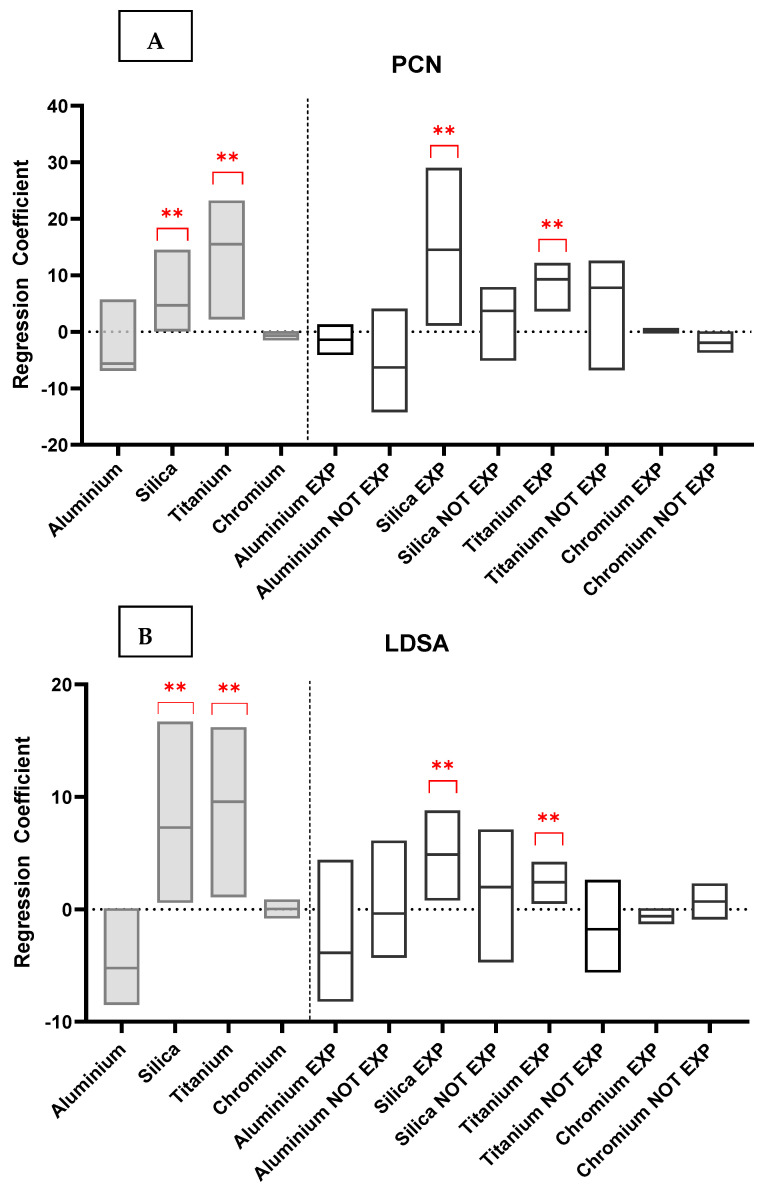
Multilevel mixed-effects models of urinary metal (dependent variables) and NM exposure metrics (PCN part **A** and LDSA part **B**) in the whole sample (left part in grey) and stratified by exposure (right part in white). Red stars show significant *p*-value.

Table 2 and Figure 2 summarize the results of the models of urinary metal and NM exposure (PCN and LDSA). The Silica and Titanium urinary concentrations increased directly and were associated with increased PCN levels (Table 2, part A; coefficient = 4.7, *p* = 0.05, 95% C.I. 0.08/4.5; coefficient = 15.5, *p* = 0.02, 95% C.I. 2.2/23.2, respectively) in the whole-sample model. Interestingly, in the models stratified by exposure, the same associations were confirmed, albeit only in the exposed subjects and only for Silica and Titanium (Table 2, part B; coefficient = 14.5, *p* = 0.03, 95% C.I. 1.1/29.03; coefficient = 9.3 *p* = 0.04, 95% C.I. 3.6/12.2, respectively). Silica and Titanium (Figure 2) were directly associated with LDSA in the whole-sample model (Table 2, part C; coefficient = 4.5, *p* = 0.04, 95% C.I. 0.6/16.7; coefficient = 11.4, *p* = 0.03, 95% C.I. 1.1/16.2, respectively). The same associations were confirmed in the models stratified by exposure (Figure 2), but only in the exposed subjects and only for Silica and Titanium (Table 2, part D; coefficient = 5, *p* = 0.04, 95% C.I. 0.8/8.8; coefficient = 2.5, *p* = 0.02, 95% C.I. 0.5/4.2, respectively).

### 3.2. Oxidative Stress

We assessed whether urinary elements were associated with OS biomarkers, and with TAP in particular; these were the only biomarkers that showed significant associations in previous studies [7,25,27].

**Figure 3:**
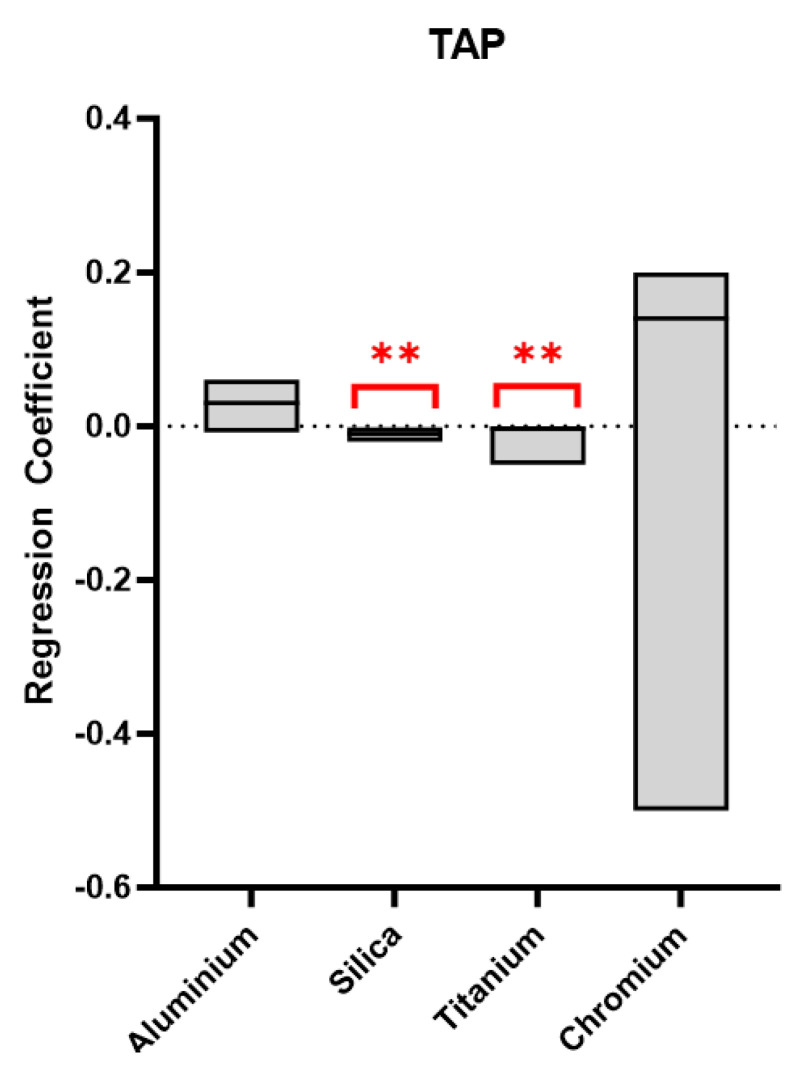
Multilevel mixed-effects models graph of TAP (dependent variable) and urinary element concentrations in the whole sample. Red stars show significant *p*-value.

Owing to the small size and the heterogeneity of the sample under study, the models were performed only in the whole sample (Figure 3). The models showed a significant decrease in TAP concentration with increasing urinary Silica and Titanium concentrations (coefficient = −0.7, *p* = 0.01, 95% C.I. −0.02/−0.002; coefficient = −0–008, *p* = 0.04, 95% C.I. −0.05/−0.0001, respectively).

### 3.3. Mediation Analysis

On the basis of the previous findings, we assessed whether metallic elements were associated with redox imbalance. Thus, we ran a raw mediation analysis between the exposure metrics (PCN and LDSA) and TAP to investigate the possible mediating role of urinary Silica and Titanium, as a proxy of NM exposure.

#### 3.3.1. Silica Mediation Analysis of Particle Metrics towards TAP

This mediation analysis was conducted to examine the mediating effect of Silica on the relationship between PCN and TAP (Table 3-part A). The total effect of the model was statistically significant (b = −4.5, z = −6.5, 95% C.I. −5.9/−3.2, *p* < 0.001). It was also found that there was a statistically significant direct effect of PCN on TAP (b = −4.8, z = −6.9, 95% C.I. −6.7/−3.5, *p* < 0.001) and a slight mediated indirect effect (b = −0.26, z = −1.5, 95% C.I. −0.7/0.05, *p* = 0.04). These results suggest that Si could account for about 1.1% of the relationship between PCN and TAP, affecting OS antioxidant defenses.

The same model was applied to examine the mediating effect of Silica between LDSA and TAP (Table 3-part B). The total effect of the model was statistically significant (b = −6.6, z = −7.02, 95% C.I. −8.4/−4.7], *p* < 0.001). The direct effect of LDSA on TAP (b = −7.1, z = −7.7, 95% C.I. −8.9/−5.3], *p* < 0.001) and the mediated indirect effect of Si were also statistically significant (b = 0.14, z = 1.9, 95% C.I. 0.04/1.03, *p* = 0.05). These results suggest that Silica could account for about 2.8% in the relationship between LDSA and TAP, possibly by decreasing OS antioxidant defenses.

#### 3.3.2. Titanium Mediation Analysis towards PCN–TAP Relationship

When assessing Titanium’s mediating effect on the relationship between PCN and TAP (Table 3-part C), the total effect and the direct effect of the model were found to be statistically significant, (total: b = −4.6, z = −6.5, 95% C.I. −5.9/−3.2, *p* < 0.001; direct (b = −4.5, z = −6.4, 95% C.I. −5.8/−3.1, *p* < 0.001, respectively). The mediating indirect effect resulted in being not statistically significant (b = −0.1, z = −1.04, 95% C.I. −0.3/0.09, *p* = 0.3). These results suggest that Ti did not contribute to the relationship between PCN and TAP.

Finally, Ti showed a statistically significant mediating effect (Table 3-part D), for both total and direct effects, on the relationship between LDSA and TAP (total: b = −6.6, z = −7.02, 95% C.I. −8.4/−4.8, *p* < 0.001; direct: b = −6.4, z = −6.9, 95% C.I. −8.3/−4.6, *p* < 0.001). However, the mediating indirect effect resulted in being not significant (b = −0.1, z = −1.06, 95% C.I. −0.4/0.1, *p* = 0.3). These results suggest that Titanium did not affect the relationship between LDSA and TAP.

## 4. Discussion

The present study assessed whether the urinary concentration of metals (Al, Ti and Cr) and a metalloid (Si) released from NMs could be reliably used in the biomonitoring of exposure to NMs. The results showed that exposed workers had higher metal urinary concentrations than the not-exposed group, especially for Ti and Si. In addition, these metals, particularly Si, mediated the relationship between NM exposure and the OS biomarkers.

Although exposure assessment for NMs has dramatically improved in recent years, relying on innovative approaches that allow the sampling in the breathing zone of workers and the translation of the aerosol characteristics into relevant metrics [28], there is still the need to assess both the internal dose and the possible effects on the target organ [8,26]. There is a general consensus regarding the likelihood that particles between 0.1 and 1.0 μm in diameter reach the lower respiratory tract, but their deposition is low compared with particles with lower and higher aerodynamic diameter due to symmetric human lung morphology [29,30]. However, the available biokinetic data suggest that the translocation rates of nanoparticles from the portal of entry—the respiratory tract—to secondary organs, is size- and charge-dependent, but the number of particles reaching the systemic circulation is actually very low [30,31]). The demonstration of the translocation of NMs from the lungs into systemic circulation is theoretically possible for metallic nanoparticles, which release metal ions or dissolve in biological media. Similarly, metallic elements are measurable in blood and urine with appropriate analytical methods, giving an estimate of current or past exposure. As a result, excretion via the kidney is also presumed to be of a low quantity, although it is possible. This limited amount can be related to the absorption of the chemical constituents of nanomaterials in the body, thus supporting the usefulness of their urinary excretion as exposure biomarkers of nanomaterials [8].

Even there is few literatures describing, the in vivo and in vitro studies confirmed that urinary metals could be useful in the working and professional exposure context. In fact, urinary metal concentrations could be a possible product of the metabolism related to NM working exposure. In addition, generally, urinary metal concentrations could be related to redox oxidative stress imbalance, resulting from their high surface-to-volume ratio and from surface characteristics such as defects of crystal structure, surface atoms with free valence electrons, and adsorbed redox-active metal ions [32,33,34].

Our results confirmed that urinary metal concentrations can be reliable biomarkers of exposure and can correctly reflect NM levels of exposure. In fact, we found that urinary metal concentrations are higher in the high-exposure subjects, and this is even more true for Si and Ti concentrations. Mediation analysis between the exposure metrics (PCN and LDSA) and TAP, carried out for urinary Si and Ti, revealed a mild, albeit significant, contribution of Si (from SiO_2_) and Ti (from TiO_2_) in the relationships between one of the exposure metrics (LDSA) and the decline in the antioxidant defense pool.

This finding seems consistent with one of the main mechanisms by which nanoparticles induce adverse health effects., i.e., the generation of ROS and oxidative stress [15]. In our study, the only statistically significant finding concerning OS was the lower values of the urinary TAP in exposed workers. TAP reflects the cumulative effects of all the antioxidants from various endogenous anti-oxidative defense systems; thus, it limits the noxious effects caused by OS. However, it is also likely that the extent of the exposure of workers did not reach levels inducing overt oxidative changes. The absence of an increase in OS biomarkers in EBC [25] and the negative TAP relationship in urine have suggested that efficient antioxidant defense mechanisms could have counterbalanced the noxious effects of metal oxides, leading to the maintenance of the redox balance in exposed workers [8,25].

The relevance of oxidative markers due to exposure to particles and fibers and especially nanomaterials has been recently reviewed [34,35]. In addition, in some cases, ions released from nanoparticles such as silver, gold, and iron can be measured in urine and in blood [7]. Biomarkers of OS and inflammation have been shown to have an association with the biopersistence of particles and fibers, resulting in frustrated phagocytosis and oxidative cellular stress, especially in the lungs [25,26,35].

Regarding the limitations of this study, the most important is linked to the exposure assessment strategy adopted. As already reported in previous studies [25,26], airborne nanoparticle concentrations were measured with a stationary device (DiSCmini), without personal exposure devices, likely underestimating exposure by inhalation.

## 5. Conclusions

Our study provides evidence that occupational exposure to air mixtures containing NMs composed of metal oxides and other dusts can increase the body burden of these foreign chemicals.

Metals released by NMs can represent an underestimated hazard for people handling technological products, increasing their body burden and potentially affecting physiological functions, e.g., the redox balance within the body. Although the health significance of such findings needs to be further elucidated, the assessment of metals in the urine of nanotechnology workers may represent a tool for estimating the body burden following long-term exposure. As a whole, a combination of biomarkers of exposure and oxidative stress can suggest early health effects, indicating the need to carry out longitudinal studies on nanotechnology workers and predictable analytical techniques, in the highest perspectives of primary prevention and health promotion. 

## Figures and Tables

**Table 1:** Descriptive statistics of the sample extracted from the cohort.

**SAMPLE**							
	**Not Exposed (*n* = 43)**		**Exposed (*n* = 40)**		**Total (*n* = 83)**		
Subjects	Male	21	Male	32	Male	53	
	Female	22	Female	8	Female	30	
BMI	Male	24.7 ± 3.4	Male	26.9 ± 4.1	Male	25.8 ± 5.8	
	Female	22.2 ± 3.2	Female	22 ± 2.3	Female	22.1 ± 2.6	
Tobacco smoke	No	39	No	28	No	67	
	Yes	4	Yes	12	Yes	16	
Alcohol	No	24	No	18	No	42	
	Yes	19	Yes	22	Yes	41	
Health score	76.6 ± 17.7		66.9 ± 21.1		70.5 ± 20		
Employment duration (years)	<5 years	30	<5 years	20	<5 years	50	
	>5 years	13	>5 years	20	>5 years	33	
PPE use	No	43	No	5	No	48	
	Yes	-	Yes	35	Yes	35	
**BIOLOGICAL BIOMARKERS**							
	**Not Exposed**		**Exposed**		**Total**		**Non-Parametric Test**
PCN [#/cm^3^]	3.7 ± 0.3		4.8 ± 0.3		4.4 ± 0.6		0.002
LDSA [µm^2^/cm^3^]	1.1 ± 0.3		1.9 ± 0.2		1.6 ± 0.5		0.003
Aluminum [µg/L]	44.7 ± 45.7		37 ± 37.9		44 ± 48.9		0.7
Silica [mg/L]	12.3 ± 5.1		19.6 ± 8.3		15.7 ± 8		0.02
Titanium [µg/L]	25.8 ± 10.1		33 ± 11.5		29.6 ± 11.4		0.03
Chromium [µg/L]	0.6 ± 0.04		0.3 ± 0.2		0.4 ± 0.5		0.3
MDA [µg/mg_CREA_]	243 ± 196		235 ± 252		240 ± 218		0.5
Isop [µg/mg_CREA_]	4.3 ± 2.8		4.1 ± 2.5		76.6 ± 17.7		0.16
TAP [µg/mg_CREA_]	1.3 ± 0.4		0.9 ± 0.3		1.01 ± 0.9		0.001

**Table 2:** Multilevel mixed-effects models of urinary metal (dependent variables) and NM exposure metrics (PCN and LDSA) in the whole sample (parts A and C) and stratified by exposure (parts B and D).

**PCN**					
Part A		**Multilevel Mixed-Effects Model (Whole Sample)**			
		**Coeff.**	**Std.Err.**	***p***	**[95% CI]**
Al		−5.6	6.2	0.08	−5.6/5.7
Si		4.7	4.8	0.05	0.08/4.5
Ti		15.5	7.2	0.02	2.2/23.2
Cr		−0.7	0.4	0.9	−1.5/0.11
Part B		**Multilevel Mixed-Effects Model (Exposure Stratified)**			
		**Coeff.**	**Std.Err.**	***p***	**[95% CI]**
Al	Exposed	−1.4	2.2	0.9	−4.1/1.3
	Not exposed	−6.3	4.8	0.3	−14.2/4.1
Si	Exposed	14.5	7.4	0.03	1.1/29.03
	Not exposed	3.7	10.9	0.7	−5.1/7.9
Ti	Exposed	9.3	11.7	0.04	3.6/12.2
	Not exposed	7.8	7.7	0.3	−6.8/12.6
Cr	Exposed	0.2	0.3	0.5	−0.3/0.7
	Not exposed	−1.9	0.9	0.4	−3.7/0.08
**LDSA**					
Part C		**Multilevel Mixed-Effects Model (Whole Sample)**			
		**Coeff.**	**Std.Err.**	***p***	**[95% CI]**
Al		−7.3	9.3	0.8	−8.4/0.11
Si		4.5	6.2	0.04	0.6/16.7
Ti		11.4	10.1	0.03	1.1/16.2
Cr		0.05	0.5	0.9	−0.8/0.9
Part D		**Multilevel Mixed-Effects Model (Exposure Stratified)**			
		**Coeff.**	**Std.Err.**	***p***	**[95% CI]**
Al	Exposed	−7.8	6.7	0.2	−8.2/4.4
	Not exposed	−2.9	4.3	0.9	−4.3/6.1
Si	Exposed	5	3.6	0.04	0.8/8.8
	Not exposed	3.5	4.4	0.4	−4.7/7.1
Ti	Exposed	2.5	1.05	0.02	0.5/4.2
	Not exposed	−2.3	3.3	0.3	−5.6/2.6
Cr	Exposed	−0.6	0.4	0.1	−1.3/0.1
	Not exposed	0.7	0.8	0.4	−0.9/2.3

**Table 3:** Mediating analysis between exposure biomarkers (PCN and LDSA) and TAP concentrations with Si (part A and B) and Ti (part C and D) as mediating factors.

**Mediation Models: SILICA**								
Part A	**PCN/Si/TAP Mediation estimates**							
**Effect**	**Label**	**Estimate**	**SE**	**Lower**	**Upper**	**Z**	***p***	**% Mediation**
Indirect	a × b	−0.26	0.169	−0.714	−0.059	−1.54	0.042	1.12
Direct	c	−4.826	0.691	−6.1798	−3.471	−6.98	<0.001	94.88
Total	c + a × b	−4.565	0.702	−5.9416	−3.189	−6.5	<0.001	100
Part B	**LDSA/Si/TAP Mediation estimates**							
**Effect**	**Label**	**Estimate**	**SE**	**Lower**	**Upper**	**Z**	***p***	**% Mediation**
Indirect	a × b	0.14	0.262	0.045	1.03	−1.96	0.05	2.78
Direct	c	−7.063	0.917	−8.86	−5.27	−7.7	<0.001	93.22
Total	c + a × b	−6.549	0.933	−8.38	−4.72	−7.02	<0.001	100
**Mediation Models: TITANIUM**								
Part C	**PCN/Ti/TAP Mediation estimates**							
**Effect**	**Label**	**Estimate**	**SE**	**Lower**	**Upper**	**Z**	***p***	**% Mediation**
Indirect	a × b	−0.101	0.0974	−0.292	0.0897	−1.04	0.298	2.22
Direct	c	−4.464	0.7019	−5.84	−3.088	−6.36	<0.001	97.78
Total	c + a × b	−4.565	0.7023	−5.942	−3.1887	−6.5	<0.001	100
Part D	**LDSA/Ti/TAP Mediation estimates**							
**Effect**	**Label**	**Estimate**	**SE**	**Lower**	**Upper**	**Z**	***p***	**% Mediation**
Indirect	a × b	−0.14	0.133	−0.4	0.12	−1.06	0.29	2.14
Direct	c	−6.409	0.934	−8.239	−4.579	−6.86	<0.001	97.86
Total	c + a × b	−6.549	0.933	−8.377	−4.722	−7.02	<0.001	100

## Data Availability

The data that were used are confidential.

## Supplementary Materials

The following supporting information can be downloaded at: https://www.mdpi.com/article/10.3390/antiox13060676/s1, Table S1: Technical information.
