# Supplementary material for: Occupational Exposure to Metal-Based Nanomaterials: A Possible Relationship between Chemical Composition and Oxidative Stress Biomarkers
Source: Antioxidants (Basel). 2024 May 31;13(6):676. doi: 10.3390/antiox13060676 (PMC11201196; doi:10.3390/antiox13060676)
Supplement: Supplementary file 1 [file antioxidants-13-00676-s001.zip › antioxidants-3004734-supplementary.pdf]

Supplementary Materials

Table S1. Technical information

Biomarker	Biomarker description	Analytical method	Limit of detection
Metals (Al, Si, Ti, Cr)	Metals concentrations proxy of NMs esposure	Inductively coupled plasma mass spectrometry (ICP-MS) (Perkin Elmer NexION 300D, Shelton, CT, USA)	Al_{27} 1 ug/l<br>Si_{29} 63 ug/l<br>Ti_{47} 0.1 ug/l<br>Cr_{52} 0.01 ug/l
Isoprostane (15-F2t-Isoprostane)	Oxidative stress	ELISA kit (EA85, Oxford biomedical)	0.08 ng/ml<br>0.1-100 ng/ml Standard curve
Malondialdehyde (MDA)	Oxidative stress	Thiobarbituric Acid Reactive Substances assay (Colorimetric) (FR40, Oxford biomedical)	72 ng/ml<br>0-20 μM MDA Standard curve
Total Antioxidant Power (TAP)	Antioxidant capacity	Cupric ion reducing antioxidant capacity assay (Colorimetric) (TA02, Oxford biomedical)	25 μg/ml<br>0-2 mM trolox Standard curve
